# Curcumin Attenuates Hyperglycemia and Inflammation in Type 2 Diabetes Mellitus: Quantitative Analysis of Randomized Controlled Trial

**DOI:** 10.3390/nu16234177

**Published:** 2024-11-30

**Authors:** Kabelo Mokgalaboni, Reneilwe G. Mashaba, Wendy N. Phoswa, Sogolo L. Lebelo

**Affiliations:** 1 Department of Life and Consumer Sciences, College of Agriculture and Environmental Sciences, Florida Campus, Roodepoort 1709, South Africa; 2 DIMAMO Population Health Research Centre, University of Limpopo, Sovenga, Private Bag X1106, Polokwane 0727, South Africa

**Keywords:** curcumin, turmeric, *Curcuma longa*, type 2 diabetes, hyperglycemia

## Abstract

Controlling hyperglycemia and inflammation in type 2 diabetes (T2D) remains an important approach to control diabetes. The use of phytochemicals found in natural herbs has been investigated widely, and there are inconsistent findings in clinical trials, likely associated with a small sample size. A meta-analysis of clinical trials was performed by conducting a comprehensive literature search on PubMed, Scopus, EBSCOHost, and Web of Sciences. The search terms included *Curcumin longa*, turmeric, curcumin, curcuma xanthorrhiza, diferuloylmethane, and type 2 diabetes. Data were analyzed using an online meta-analysis tool, Jamovi version 2.4.8 and IBM SPSS statistics version 29. The data were reported as either mean difference (MD) or standard mean difference (SMD) and 95% confidence intervals. The evidence from 18 trials with 1382 T2D with a mean age of 55.9 years was analyzed. Supplementation with curcumin led to a significant decrease in fasting blood glucose, MD = −11.48 mg/dL, 95%CI (−14.26, −8.70), *p* < 0.01 and glycated hemoglobin, MD = −0.54%, 95%CI (−0.73, −0.35), *p* < 0.01. Additionally, there was a significant decrease in C-Reactive Protein in curcumin compared to a placebo, SMD = −0.59, 95%CI (−1.11, −0.07), *p* = 0.03. The findings observed in this study suggest that curcumin can ameliorate hyperglycemia and inflammation in T2D compared to a placebo. While the potential benefits were observed, it is recommended that future trials focus on finding a suitable dose and duration of intervention and incorporate formulation in curcumin to enhance its absorption.

## 1. Introduction

Type 2 diabetes (T2D) is defined by the World Health Organization (WHO) as a metabolic condition characterized by high blood glucose due to insulin insufficiency or resistance [1]. The prevalence of diabetes is alarmingly high; globally, it was 10.5% in 2021, and it is estimated that this figure will reach 12.2% by 2040 [2]. People living with T2D are at risk of various complications, such as anemia [3], cardiovascular disease (CVD), and atherosclerosis [4,5]. More recently, CVD complications have been deemed as contributing factors to mortality among T2D [6]. Hyperglycemia, as observed in T2D, is associated with inflammation characterized by elevated levels of proinflammatory cytokines, such as interleukin-6 (IL-6), tumor necrosis factor-alpha (TNF-α), and C-reactive protein (CRP) and these altogether increase the risk of CVD [7,8]. Therefore, controlling hyperglycemia and inflammation in T2D is important to attenuate associated complications. T2D patients have long relied on antihyperglycemic drugs, such as sulfonylureas [9] and biguanides [10], which are well-established antihyperglycemic drugs. However, these drugs are associated with some adverse effects. Biguanides such as metformin are associated with hypoglycemia, lactic acidosis, and vitamin B_12_ deficiency, predisposing patients to a wide range of complications [11,12,13]. Sulfonylureas are also associated with hypoglycemia, weight gain, and an increased risk of cardiovascular events [14,15,16]. On the other hand, herbal remedies have also been used to control and manage hyperglycemia in T2D [17]. Our team has recently explored the antihyperglycemic effect of okra (*Abelmoschus esculentus* L.) in T2D [18]. Although its beneficial effect was observed on fasting blood glucose (FBG), no effect was noted on glycated hemoglobin (HbA1C), suggesting its potential limitation. Therefore, other herbal remedies must be explored in T2D. Another medicinal remedy used for its beneficial properties is *Curcuma longa* [19,20,21]. *Curcuma longa* belongs to the family of Zingiberaceae [22], and it produces the active compound curcumin, which contains polyphenols necessary for good health [22,23]. Curcumin has been confirmed to possess anti-inflammatory and antioxidant properties in T2D [24,25]. While its anti-hyperglycemic effects are observed in animal models of diabetes [26,27], the benefits in controlling hyperglycemia and inflammation among individuals living with T2D are inconsistent. Other trials suggest no effect on FBG and HbA1C as markers of hyperglycemia [28,29,30] or CRP as a proinflammatory marker [29,31]. The above-mentioned controversy shows the potential limitation of curcumin in T2D. Therefore, in this study, we sought to investigate the antihyperglycemic effect of curcumin in T2D. Furthermore, we explore the effect of curcumin on CRP as a marker of inflammation.

## 2. Materials and Methods

This study followed the updated guidelines of systematic reviews and meta-analysis (PRISMA) [32] and Cochrane guidelines [33]. It also adhered to the adjusted PICOS criteria [34], which outline the population (P), intervention (I), comparator (C), outcomes (O), and study design (S). PICO was as follows: adult patients living with T2D, treated with curcumin/turmeric, placebo was a comparator, and the outcomes were the changes in FBG, HbA1C, and CRP. The protocol supporting this work was prospectively registered (Reference number: CRD420246030023). The results are reported using the PRISMA checklist (Supplementary File S1).

### 2.1. Literature Search and Search Strategy

Independent researchers (KM and WNP) conducted a comprehensive search on PubMed, Scopus, EBSCOhost, and Web of Science, following the predefined PICO criteria. A third researcher (SL) was involved in arbitration in case of disagreement. The search terms used included *Curcuma longa*, curcumin, *Curcuma* xanthorrhiza, turmeric, and type 2 diabetes and were adapted for the four databases using the two Boolean operators (“OR” and “AND”). No language restrictions were applied to the search. The searches on PubMed, EBSCOhost, and Web of Sciences were restricted to randomized controlled trials (RCTs). The exact search strategy is presented in Supplementary File S2 and Table S1 of Supplementary File S3.

### 2.2. Risk of Bias (ROB), Quality of Trials, and Certainty of Evidence

Two researchers assessed the risk of bias using the Cochrane risk of bias tool [35] and visualized it using the ROB visualizing tool [36]. The following domains were considered: (D1) bias due to randomization, (D2) bias due to deviation from intended intervention, (D3) bias due to missing data, (D4) bias due to outcome assessment, and (D5) bias due to selection of reported results. The overall quality of individual trials was judged based on the level of bias across different domains. Briefly, the trial was regarded as good quality when all domains were low risk or when one domain was judged as having some concerns. The trial was classified as low quality if it had at least one high risk in one domain. Certainty of evidence was evaluated following grading of recommendation, assessment, development, and evaluation (GRADE) [37]. This approach considers five domains: risk of bias, inconsistency, indirectness, and imprecision. The evidence was upgraded based on the large effect size, plausible confounding, or dose-dependent relationship. The GRADEpro https://www.gradepro.org/ (accessed on 9 October 2024) was used to evaluate certainty, and the results were interpreted as per an upgraded previous guideline [38]. The overall certainty was classified as either low, very low, moderate, or good evidence and presented as a summary of the findings table.

### 2.3. Eligibility Criteria and Selection Process

Microsoft Excel and Mendeley Reference Manager (Version 2.126.0) were used to facilitate the screening process, remove duplicates, and store all relevant trials. All trials were screened by two independent researchers (KM and WNP). The initial screening was based on the titles, abstracts, and keywords. The full texts of retrieved trials were included in the final analysis if they met the predefined PICO criteria: (i) the trials in which the population was adult living with T2D, (ii) trials that used curcumin or turmeric as an intervention, (iii) trials with the control group as a placebo, and (iv) trials that reported change in FGB, HbA1C, and CRP (baseline and post-treatment data). Any studies conducted on patients other than those with T2D, studies without control, preclinical studies, reviews, study protocols, and studies that used treatment other than curcumin were excluded. Studies that reported no changes in FGB, HbA1C, and CRP, letters to the editor, insufficient data, trials using the same data, and sample size from another trial reporting the same parameters were excluded.

### 2.4. Data Extraction, Items, and Handling

Two independent researchers (KM and RGM) independently extracted data from each trial. The third independent researcher checked the data for consistency and to avoid the risk of bias in the process of extraction. Among the items extracted were the authors’ names, year of publication, study design, participant’s conditions, sample size (number of patients with the condition, those on curcumin and placebo), dose and duration of intervention, baseline age and body mass index in both groups, gender distribution in both groups, changes in the level of FBG, HbA1C, and CRP in both groups. The data were further shared with the other researchers (WNP and SLL) for independent adjudication. Mean values and standard deviations (SDs) were extracted from each study; in case median and range were reported, the website http://vassarstats.net/median_range.html (accessed 28 September 2024) was used to estimate mean and SD. If the standard error of the mean (SEM) was provided, SD was calculated using the formula, $SEM=\frac{SD}{\sqrt{n}}$ . On the other hand, SD was also estimated using the formula, $SD=\frac{IQR}{1.35}$ when interquartile range (IQR) was reported. In accordance with the Cochrane Handbook, the change (mean and SD) in FBG, HbA1C, CRP were used to calculate the overall effect estimates. If the change in mean and SD was not reported from the trial, the following formula was used to estimate the mean difference (mean final–mean baseline). The change in SD was calculated using the formula, ∆ SD=(√(SDf^2+SD^2−2(r∗SDf+SDb) . The correlation coefficient (r) of 0.5 was used [39]. The SDf and b are defined as standard deviations for post-treatment and before-treatment, respectively. Fasting blood glucose data reported as mmol/L were converted to mg/dL for consistency with the rest of the trials using the following calculator: https://www.mdapp.co/blood-sugar-conversion-calculator-71/ (accessed 7 October 2024).

### 2.5. Data Analysis and Reporting

Meta-analysis was conducted when more than two trials assessed the same parameters. The meta-analysis online tool was used to analyze data and to generate the plots: https://metaanalysisonline.com/ (accessed 7 October 2024), Jamovi (version 2.4.8) and IBM SPSS statistics (version 29) were used to perform meta-regression and subgroup analysis. The effect estimates were reported as either mean difference (MD) or standardized mean difference (SMD) and 95% CI and were presented using forest plots. For SMD values, Cohen’s d estimation was used to classify the effect size (0.2, 05, and 0.8 were classified as small, medium, and large, respectively). Publication bias was visualized through funnel plots and statistically with the Eggers test [40,41]. Sensitivity was assessed by excluding one study at a time and re-evaluating the effect size [42]. Heterogeneity was assessed using I^2^ statistic, where an I^2^ ˃ 50% prompted further analysis through subgroup analysis and meta-regression to find the association between different confounding factors and the outcomes. A two-tailed *p*-value of <0.05 was considered to be statistically significant.

## 3. Results

### 3.1. The Results of the Search and Trials Section

**Figure 1:**
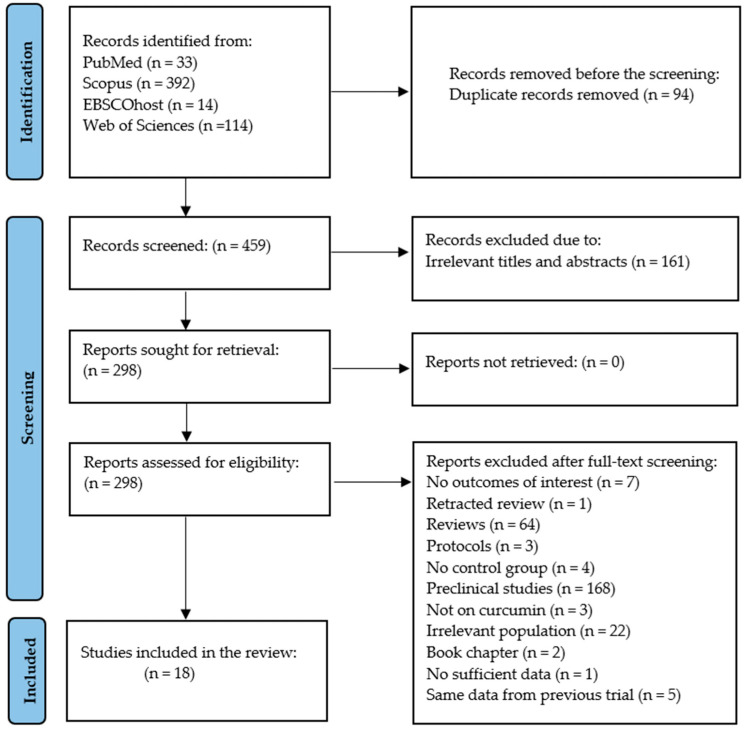
Preferred reporting items for systematic review and meta-analysis (PRISMA) flow diagram.

Evidence from PubMed, 145 Scopus, EBSCOhost, and Web of Sciences yielded five hundred and fifty-three records. The results from both databases were combined on a Microsoft Excel sheet for screening; of these 553 records, 94 were identified as duplicates and were removed (Supplementary File S3). Screening of the title and abstract revealed that 161 were irrelevant records in this phase (Supplementary File S2). Among the records that were screened for eligibility, 280 were excluded for specific reasons that included review articles, retracted review, study protocols, letters to the editor, book chapter, studies without outcomes of interest, and studies without a control group. Other exclusions were due to irrelevant populations (healthy, diabetic nephropathy, neuropathy, obesity, diabetic macula), preclinical studies (in vitro, in vivo, and study in dogs), studies without relevant treatment, studies with no sufficient data for meta-analysis, the same trial reported by the same author using the same data and sample size. Finally, only 18 trials were found relevant, satisfying the PICO criteria, and further included in the analysis [28,29,30,31,43,44,45,46,47,48,49,50,51,52,53,54,55,56] (Figure 1).

### 3.2. Individual Trial Risk of Bias Assessment

**Figure 2:**
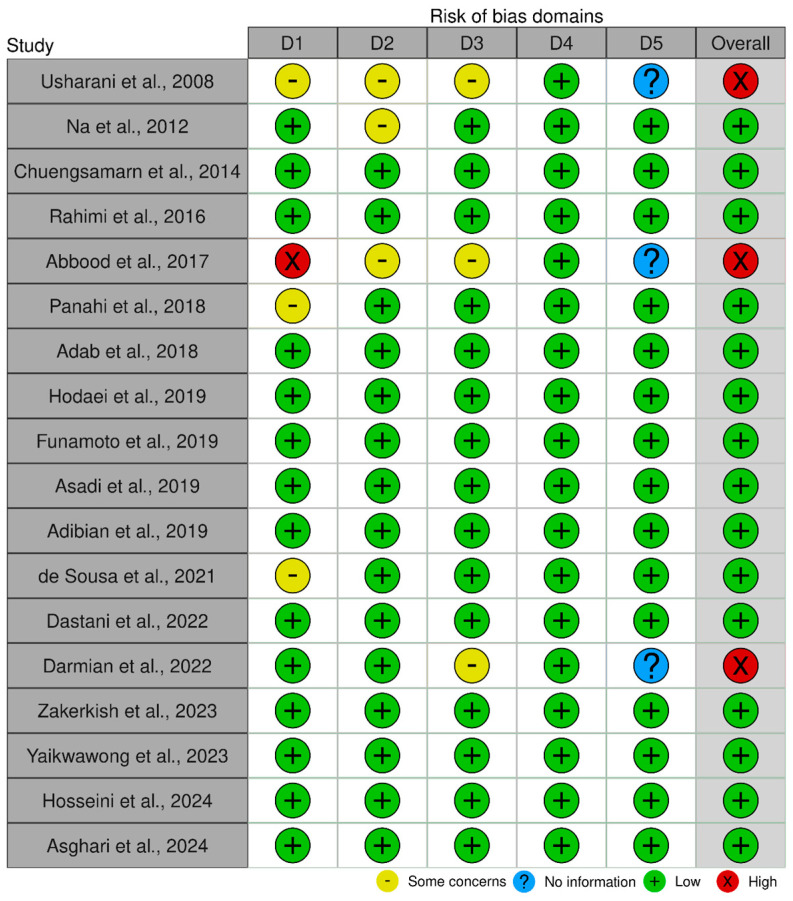
Individual risk of bias (ROS) and individual domains across the included trials [28,29,30,31,43,44,45,46,47,48,49,50,51,52,53,54,55,56].

The summary of the risk of bias in individual trials is presented in Figure 2. All domains (D1–D5) were assessed in all trials. For the first domain (randomization process), only 14 trials reported the methods of randomization they employed, and thus, they were judged as low risk. However, despite being randomized controlled trials, four trials did not state the process of randomization and thus were judged as having some concerns, while one was judged as having no information about randomization. For domain 2, due to the lack of registrations of the trials or publication of the protocols prior to commencement, this was judged as having some concerns in at least three trials.

For selective reporting bias, 15 trials registered their protocols on registry bodies, and no deviation was observed from the intended outcomes, and they were judged as low risk. However, three trials [43,53,55] did not register their protocols and thus were judged to have an unclear risk of bias and information, respectively. No conclusion could be made about deviation from the originally planned measures without published protocols or trial registration (Figure 2). Overall, the quality of the included trials was good in 15 (83%) of the trials [29,30,31,43,44,45,46,47,48,50,51,52,56], as all domains were rated low risk or with concern in only one domain and poor in 3 (16%) of the trials [28,49,53,54,55] where one or more domains were judged as high risk (Figure 2).

### 3.3. Description of Included Trials

Eighteen trials [28,29,30,31,43,44,45,46,47,48,49,50,51,52,53,54,55,56] published between 2008 and 2024 were deemed relevant according to the preplanned PICO criteria for this study. The trials were conducted across six countries: one from Brazil [54] and China [56], eleven from Iran [31,43,44,45,46,47,48,49,50,51,52,53], and one each from India [28], Iraq [55], Japan [29], and Thailand [30]. All trials were double-blinded except for two parallel trials [28,29] and a single-blinded randomized trial by Darmian [53]. Only three trials included T2D with hyperlipidemia [31,53,55], while the remaining had T2D only. The overall sample size was 1382 T2D and hyperlipidemia in these 18 trials. The sample size was 693 for the curcumin and 689 for the placebo group. Although one trial did not report gender distribution, 480 T2D were males, and 870 were females among all analyzed trials. All studies reported baseline BMI except for three trials [44,53,55]. The overall mean BMI for the curcumin group was 27.85 ± 3.83 kg/m^2^, and for the placebo group was 27.60 ± 3.61 kg/m^2^ (Table 1). The dosages of curcumin ranged from 80 mg to 2100 mg per day. The duration of intervention ranged from as short as 8 weeks (2 months) to approximately 52 weeks (12 months). The average age in both groups was 55.90 years (Table 1).

### 3.4. Quantitative Analysis

#### 3.4.1. The Effect of Curcumin on Fasting Blood Glucose

**Figure 3:**
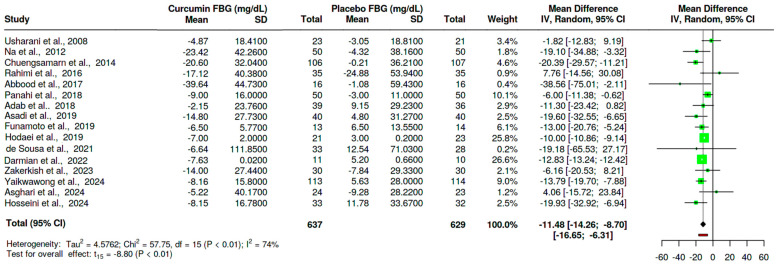
Effect of curcumin on FBG in T2D [28,29,30,31,43,45,46,47,48,49,50,52,53,54,55,56]. The black-shaped diamond represents the overall effect estimate, the green square shows the sample size of the individual trials, and the error bars show individual trial confidence intervals. SD: standard deviation, CI: confidence intervals, IV: inverse variance.

Sixteen trials [28,29,30,31,43,45,46,47,48,49,50,52,53,54,55,56] assessed the effect of curcumin on fasting blood glucose in T2D. These trials had equivalent sample sizes, curcumin (n = 637) and placebo (n = 629). The overall evidence showed a statistically significant reduction in FBG in the T2D group from curcumin compared to placebo MD = −11.48 mg/dL, 95%CI (−14.26, −8.70), *p* < 0.01 (Figure 3). However, there was evidence of heterogeneity (I^2^ = 74%, *p* < 0.01).

#### 3.4.2. Effect of Curcumin on Glycated Hemoglobin in T2D Patients

**Figure 4:**
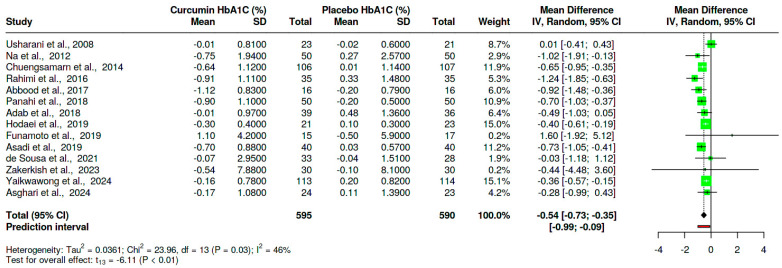
Effect of curcumin versus placebo on HBA1C in T2D patients [28,29,30,43,45,46,47,48,49,50,52,54,55,56]. The black-shaped diamond represents the overall effect estimate, the green square shows the sample size of the individual trials, and the error bars show individual trial confidence intervals. SD: standard deviation, std: standardized mean difference, CI: confidence intervals, IV: inverse variance.

Fourteen trials [28,29,30,43,45,46,47,48,49,50,52,54,55,56] that assessed the effect of curcumin on glycated hemoglobin in comparison with placebo were analyzed in this study. These trials had sample sizes of 595 and 590 for the curcumin and placebo groups, respectively. The overall results of this meta-analysis revealed a significant reduction in HbA1C, MD = −0.54, 95%CI (−0.73, −0.35), *p* < 0.01. It is worth noting that there was moderate heterogeneity (I^2^ = 46%, *p* = 0.03) (Figure 4).

#### 3.4.3. The Effect of Curcumin on C-Reactive Protein

**Figure 5:**
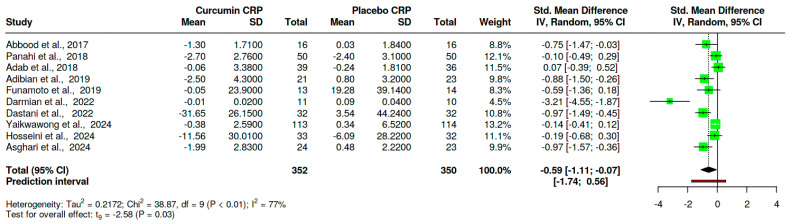
Effect of curcumin on CRP in T2D patients [29,31,44,45,47,48,49,51,53,55]. The diamond shape represents the overall effect size, the square shows the sample size of the individual trials, and the error bars show individual trial confidence intervals, SD: standard deviation, std: standardized, CI: confidence intervals, IV: inverse variance, and CRP: C-reactive protein.

Ten trials [29,31,44,45,47,48,49,51,53,55] assessed the effect of curcumin on CRP in T2D compared to placebo. The sample size was 352 and 350 for the curcumin and placebo groups, respectively. The overall effect estimates from the meta-analysis demonstrated a significant reduction in CRP in curcumin compared to placebo, SMD = −0.59, 95%CI (−1.11, −0.07), *p* = 0.03. Of concern was a high level of heterogeneity (I^2^ = 77%, *p* < 0.01) (Figure 5).

### 3.5. Sensitivity Analysis

For FBG, excluding one study at a time showed that removing the following trials [43,53] led to a significant change in effect size after re-analysis. Removal of the Darmien et al., 2022 [53] study changed the effect size from the original to MD = −11.28 mg/dL, 95%CI (−14.45, −8.12), *p* = 0.000, while for Hodaei et al., 2019 [43] study, MD = −11.93 mg/dL, 95%CI (−15.05, −8.80), *p* = 0.000. For HbA1C, no change was observed following the exclusion of trials one by one, confirming the stability of our findings. However, excluding the trial by Darmian et al., 2022 [53] for CRP, led to a significant change in the effect size with SMD = −0.44, 95%CI (−1.04, −0.16), *p* = 0.002.

### 3.6. Assessment of Association Between Moderators and Effect Size

**Figure 6:**
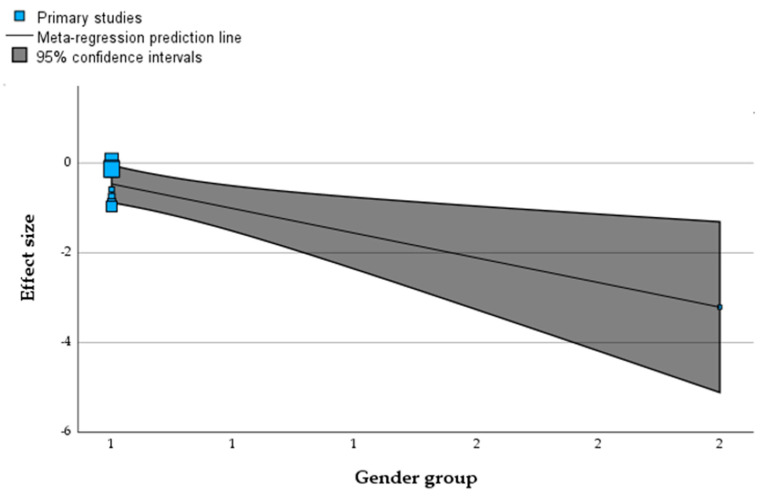
Meta-regression, showing an association between gender and CRP. Square blocks show the number of trials, and the line shows the meta-regression.

Meta-regression analysis demonstrated no significant association between age, gender, patient condition, dose of curcumin, duration of intervention, or quality of the trial with a reduction in FBG or HbA1C (*p* > 0.05) (Table 2). However, gender was deemed a significant moderator associated with reduced CRP (*p* < 0.05) (Table 2). Figure 6 shows a significant association between CRP and gender.

### 3.7. Subgroup Analysis

In this study, a subgroup was conducted to explore the source of heterogeneity. The FBG, HbA1C, and CRP results are presented in Table S2 of Supplementary File S3. In brief, the results showed that, for FBG, the inclusion of both T2D and those with hyperlipidemia may have contributed to heterogeneity. On the other hand, for HbA1C, the age of the participants may have introduced heterogeneity as patients below and above 50 were included, and subgroup analysis revealed zero heterogeneity among patients younger than 50 and a substantial reduction in heterogeneity in those above 50 years. For CRP, although there was a fluctuation in heterogeneity levels, this was minimal (60.9 versus 77%). In some cases, the heterogeneity was increased to 90.1% higher than the initial level; this suggests the observed variation was not due to any of the factors in this study.

### 3.8. Assessment of Publication Bias Across the Included Trials

**Figure 7:**
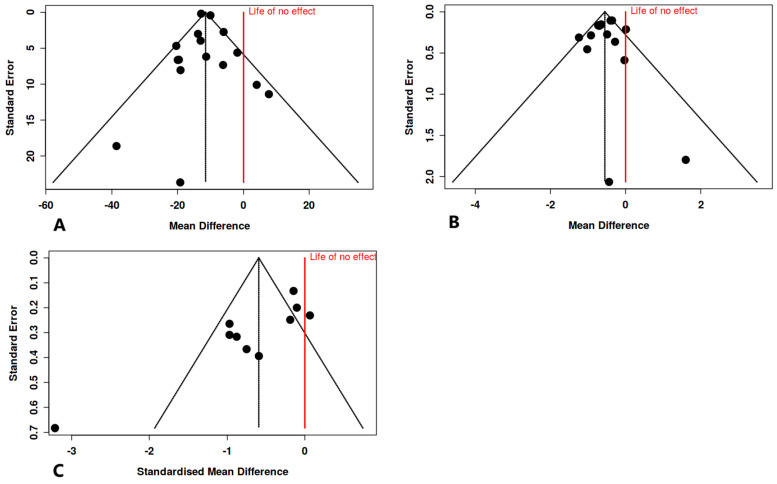
Funnel plots depicting publication bias across the analyzed trials. (**A**) fasting blood glucose [28,29,31,43,45,46,47,48,49,50,52,53,54,55,56]; (**B**) glycated hemoglobin [28,29,43,45,46,47,48,49,50,52,54,55,56]; (**C**) C-reactive protein [29,31,44,45,47,48,49,51,53,55].

For FGB, the funnel plot did not reveal any potential publication bias (Figure 7A). The Egger’s test does not support the presence of funnel plot asymmetry (intercept: 0.31, 95% CI: −0.79, 1.4, t: 0.546, *p* = 0.594). Similarly, for the HbA1C, the funnel plot revealed no potential publication bias (Figure 7B). This was supported by Egger’s test, which showed no presence of funnel plot asymmetry (intercept: −0.27, 95% CI: −1.63,1.08, t: −0.396, *p* = 0.699). However, for CRP, the funnel plot demonstrated a potential publication bias (Figure 7C). This was confirmed by the results of Egger’s test, which supports the presence of funnel plot asymmetry (intercept: −4.07, 95% CI: −6.25, −1.89, t: −3.663, *p* = 0.006).

### 3.9. Certainty of Evidence Included in the Analysis

The certainty of evidence based on GRADE is presented as a summary of the finding table for the outcomes as shown in Table 3. Briefly, for FBG, the evidence was low, and this was downgraded by the risk of bias, inconsistency, and impression and upgraded by plausible confounding. For HbA1C, the evidence was downgraded due to the risk of bias, and thus, overall evidence was classified as moderate certainty. The evidence was classified as low for CRP, as evidence was downgraded based on the risk of bias, indirectness, and publication bias.

## 4. Discussion

The prevalence of T2D is alarming worldwide, and this contributes to global mortality. The economic burden of its complications is worse in developing countries where access to proper antihyperglycemic medication is limited [57,58]. The current study synthesized evidence from clinical trials to evaluate the potential benefits of curcumin on hyperglycemia and CRP in T2D patients. This study reports that curcumin supplementation in T2D ameliorates hyperglycemia, as demonstrated by reduced fasting blood glucose and glycated hemoglobin. Additionally, curcumin alleviated inflammation, as shown by reduced CRP. Meta-regression showed that gender was substantially associated with a decrease in HbA1C. As T2D is characterized by hyperglycemia and inflammation in T2D, its progression is associated with myriads of complications, including CVD. Therefore, controlling hyperglycemia and inflammation in T2D can better prevent or minimize the associated complications. As demonstrated in this study, the administration of curcumin in T2D can be considered a reliable alternative therapeutic agent in managing T2D.

While the effects were evident in this study, other studies had reported conflicting findings. A trial by [43] showed no effect of curcumin on HbA1C in T2D post-curcumin treatment; this was consistent when curcumin was compared to a placebo. Additionally, curcumin had no significant effect on FBG at baseline and after ten weeks of curcumin treatment compared to a placebo in the same trial. While no effect of curcumin was observed after 3 months, the administration ofcurcumin at6, 9, and 12 months significantly decreased FBG [48]. This suggests that the duration of intervention may play a significant role in the efficacy of curcumin. Consistent with our findings is the evidence from a rodent model of diabetes, which has confirmed the antihyperglycemic effect of 400 mg of curcumin. More recently, [31] also reported a significant reduction in FBG after 12 weeks of 505 mg curcumin treatment in T2D patients, thus supporting our findings. Although the benefits of curcumin are acknowledged, the potential mode of action is still a research area for discussion. Other researchers suggest that curcumin can exert its antihyperglycemic effect by inhibiting glucose-6-phosphatase and phosphoenolpyruvate carboxykinase, which are involved in gluconeogenesis in the liver [59,60]. This subsequently leads to reduced blood glucose levels. It upregulates the expression of glucose transporters, including (GLUT-2, 3, and 4) in the muscle and adipose tissue [61,62]. This enhances the movement of glucose from the blood into the cells, thus reducing blood glucose. As T2D is associated with impaired carbohydrate or glucose metabolism, it is assumed that the level of enzyme α-amylase and α-glucosidase that catalyzes carbohydrates into glucose molecules may be elevated [63]. Therefore, the impact of curcumin on such enzymes may be important in regulating glucose metabolism and controlling hyperglycemia. Other studies have proposed that curcumin may exert its antihyperglycemic potential by inhibiting the function of both α-amylase and glucosidases [64,65].

Chronic inflammation is associated with hyperglycemia, a critical feature among T2D patients [8]. The effect of curcumin on CRP was observed, supported by evidence from other trials included in this meta-analysis [44,51,53,55]. Interestingly, Dastani et al., 2022 [51] used a formulation of nano-curcumin, and this trial revealed a substantial effect on CRP. This suggests that the use of a curcumin formulation can enhance the absorption of curcumin in T2D. Despite the notable effect of curcumin on CRP, eight weeks of treatment with 21,000 mg of turmeric showed no effect on CRP in T2D [45]. This support previous trials [28,29]. Similarly, the new evidence from another clinical trial also demonstrated no effect of 500 mg of curcumin on CRP [31]. While the effect was not observed in the above trial, it is important to note that this trial used a combination treatment with piperine as a formulation to enhance the absorption of curcumin. This trial raises the question of the efficacy of the formulation in the improvement in curcumin absorption, especially regarding its efficacy in reducing CRP. It has been reported that piperine, compared to other forms of formulations, increases curcumin absorption by 1.5-fold lower than hydrophilic careers (45.9-fold) and nanoparticles by 185-fold [66,67,68]. Secondly, the participants investigated had a coexistence of T2D with hypertriglyceridemia. All of these factors might have contributed to the observed potential limitation. Therefore, it is important to understand the mechanism by which curcumin ameliorates inflammation. One important proposed mechanism is the downregulation of the expression of nuclear factor kappa-light-chain-beta cells (NF-κβ) [69,70]. NF-κβ contribute to inflammation by activating the expression of proinflammatory genes in T2D [71].

On the other hand, curcumin seems to activate the AMP-activated protein kinase (AMPK) pathway, which also downregulates NF-κβ [72,73]. The suppression of NF-κβ subsequently inhibits the production of associated inflammatory cytokines, such as TNF-α, IL-6, and CRP [74,75]. Curcumin also inhibits the activity of inhibitory kappa beta (Iκβ) kinase, which prevents the phosphorylation and degradation of Iκβα [76]. This further prevents translation of NF-κβ and suppresses their role in the activation of proinflammatory genes and proteins [74,75]. Thereby ameliorating inflammation and complications thereof. As curcumin has proven to downregulate the expression of the NF-κβ pathway, its effect on this pathway is important in regulating inflammation. On the other hand, curcumin reduces inflammation by inhibiting the production of nitric oxide and inducible nitric oxide synthase (iNOS), which are associated with the downregulation of NF-κβ and activator protein-1 (AP-1) [77,78,79]. Curcumin exerts its NO-lowering effects by scavenging the free reactive oxygen species or reducing the iNOS activity [77,78]. The cyclooxygenase (COX) pathway is involved in inflammation [80]. Its downregulation may serve as a target in ameliorating inflammation. Inhibition of cyclooxygenase is reportedly associated with reduced CRP [81]. Interestingly, curcumin also downregulates COX, inhibiting the expression of NF-κβ and reducing CRP [82,83].

The current study used four main databases to retrieve evidence, which was conducted independently to minimize the risk of bias. This rigorous and comprehensive search from different databases ensured that all relevant trials were identified. This study assessed evidence from RCT classified as high-quality evidence in the research community. While it is important to acknowledge that 3 trials (17%) analyzed in this study were classified as poor quality, the overall quality of 15 (83%) trials was deemed good in accordance with Cochrane’s risk of bias guidelines. The GRADE was also used, classifying some evidence as moderate and some as having low certainty. While heterogeneity was observed, random effect model meta-analysis, meta-regression, and subgroup analysis were undertaken, and only gender was found to be associated with a change in CRP. The current study had a sufficient sample size (1382) with improved statistical power compared to individual trials. Publication bias was only observed for evidence that assessed CRP, possibly due to the low reporting of negative findings in different trials. While the evidence gathered in this study suggests the potential benefits of curcumin on hyperglycemia and inflammation, the evidence was only distributed in a few countries, and none was obtained from African countries where the prevalence of T2D is very high.

## 5. Conclusions and Future Directions

Hyperglycemia and inflammation are prevalent features in T2D, and they contribute to myriads of complications, including CVD. However, the results obtained from this study demonstrated a protective effect of curcumin against both hyperglycemia and inflammation in T2D and those coupled with hyperlipidemia. Therefore, *Curcuma longa* and its active compound, curcumin, can improve hyperglycemia and inflammation by reducing fasting blood glucose, glycated hemoglobin, and C-reactive protein. Further clinical trials are warranted to evaluate the effect of curcumin on additional inflammatory markers, associated genes, and protein expression to ascertain its effect on inflammatory pathways. The obtained results suggest that T2D patients can use curcumin as a supplement to reduce hyperglycemia and inflammation. Hence, curcumin can be used as a supplement to the diet of patients living with T2D to curb hyperglycemic-associated complications. While the available evidence from that study shows the potential benefits of curcumin in T2D, it is worth noting that different doses and durations of intervention were employed, which makes it difficult to identify the exact dose that can be used as antihyperglycemic and anti-inflammatory. The use of formulations is essential in improving the bioavailability and absorption of curcumin. Therefore, we recommend that future trials focus on the potential effective dose and the ideal duration of intervention on a large scale of individuals living with T2D. Additionally, the incorporation of curcumin formulations would improve the overall effect of curcumin through enhancement of its absorption and bioavailability of curcumin.

## Figures and Tables

**Table 1:** General information about the effects of curcumin in type 2 diabetes.

Author	Study Design	Country	Participants State	Sample Size	Intervention	Age	BMI	Gender Distribution n (%)	Summary of Findings
Abbood et al., 2017 [55]	Randomized parallel trial	Iraq	Type 2 diabetes (T2D) and hyperlipidemia	Curcumin (n = 16) Placebo (n = 16)	Curcumin 500 mg or 50 mg of placebo twice a day for 12 weeks.	46.76 ± 7.89 46.76 ± 7.89	NR	NR	Curcumin significantly reduced FBG, HbA1C, and serum CRP.
Adab et al., 2018 [45]	Randomized, double-blinded clinical trial	Iran	T2D	Turmeric (n = 39) Placebo (n = 36)	Three 700 mg turmeric capsules, placebo group received three placebo capsules for eight weeks.	54.76 ± 6.00 55.66 ± 8.64	28.98 ± 3.68 28.82 ± 4.96	19 (48.7) 17 (47.2)	Turmeric showed no effect on FBG, HbA1C, and CRP.
Adibian et al., 2019 [44]	Randomized, double-blinded, placebo-controlled trial.	Iran	T2D	Curcumin (n = 21) Placebo (n = 23)	500 mg curcumin capsules three times per day or placebo for ten weeks.	58 ± NR 60 ± 7	NR	13 (61.6) 9 (39.1)	Curcumin decreased CRP.
Asadi et al., 2019 [46]	Randomized, double-blinded, parallel, placebo-controlled clinical trial	Iran	T2D	Nano-curcumin (n = 40) Placebo (n = 40)	One 80 mg capsule of nano-curcumin or placebo for eight weeks.	53.3 ± 6.5 54.6 ± 6.2	31.1 ± 4.2 30.8 ± 3.8	5 (12.5) 5 (12.5)	Curcumin decreased FBG and HbA1C.
Asghari et al., 2024 [47]	Randomized double-blinded clinical trial	Iran	T2D	Curcumin (n = 25) Placebo (n = 25)	80 mg nano curcumin and two capsules containing a placebo of ω − 3 Fatty Acids for 12 weeks.	54.56 ± 8.30 57.48 ± 11.27	27.78 ± 2.15 27.82 ± 1.73	12 (48) 11 (44)	Nano Curcumin decreased FBG, HbA1C, and CRP.
Funamoto et al., 2019 [29]	Randomized, double-blind, parallel, placebo-controlled trial	Japan	T2D	Curcumin (n = 15) Placebo (n = 17)	180 mg of Theracurcumin capsule for six months.	70 ± 6 69 ± 7	24.9 ± 4.6 25.0 ± 2.6	9 (60) 13 (76.5)	No effect on FBG, HbAC, and CRP.
Panahi et al., 2018 [49]	Randomized double-blind placebo-controlled trial	Iran	T2D	Curcumin (n = 50) Placebo (n = 50)	Curcuminoids (500 mg curcumin plus 5 mg piperine) or placebo for three months.	43 ± 8 41 ± 7	26 ± 2 27 ± 2	25 (50) 26 (52)	Curcumin decreased FBG and HbA1C without effect on CRP.
Zakerkish et al., 2023 [50]	Randomized, double-blind, controlled trial	Iran	T2D	Curcumex (n = 30) Placebo (n =30)	Curcumex capsule (320 mg) twice a day or placebo every 12 h for 90 days.	58.27 ± 9.83 52.07 ± 8.84	29.37 ± 5.28 28.25 ± 4.22	8 (26.67) 12 (40)	Curcumin significantly decreased FBG and HbA1C.
Rahimi et al., 2016 [52]	Double-blind randomized placebo-control add-on clinical trial	Iran	T2D	Curcumin (n = 35) Placebo (n = 35)	Nano-curcumin (as nano-micelle 80 mg/day) or placebo for three months.	56.34 ± 11.17 60.95 ± 10.77	26.92 ± 2.71 27.27 ± 3.59	17 (48.5) 14 (40)	Curcumin significantly decreased FBG and HbA1C.
Na et al., 2013 [56]	Randomized, double-blind, placebo-controlled trial	China	T2D	Curcumin (n = 50) Placebo (n = 50)	150 mg curcuminoids or 150 mg placebo capsule twice daily for three months.	55.42 ± 6.40 54.72 ± 8.34	27.12 ± 2.26 27.42 ± 3.04	24 (48) 25 (50)	Curcumin significantly decreased FBG and HbA1C.
Hodaei et al., 2019 [43]	Randomized, double-blinded, placebo-controlled trial	Iran	T2D	Curcumin (n= 21) Placebo (n = 23)	Three capsules of 500 mg of curcumin and a placebo for ten weeks.	58 ± 8 60 ± 7	29.2 ± 3.76 28.2 ± 2.5	13 (61.6) 9 (39.1)	Curcumin significantly decreased FBG without effect on HbA1C.
Usharani et al., 2008 [28]	Randomized, parallel-group, placebo-controlled trial	India	T2D	Curcumin (n = 23) Placebo (n = 21)	Two 150 mg of curcumin capsules or one placebo capsule twice daily for eight weeks.	55.52 ± 10.76 49.75 ± 8.18	24.66 ± 2.42 23.98 ± 2.35	12 (52.17) 11(52.38)	No effect on FBG and HbA1C.
Chuengsamarn et al., 2014 [30]	simple randomized, double-blinded, placebo-controlled trial	Thailand	T2D	Curcumin (n = 106) Placebo (n = 107)	Three capsules of 250 mg curcumin or placebo twice a day for six months.	59.16 ±10.89 59.58 ± 10.76	27.09 ±5.35 26.84 ±4.34	50 (46.7) 47 (44.3)	No effect on FBG and HbA1C.
Darmian et al., 2022 [53]	Single-blind, randomized, placebo-controlled study	Iran	Hyperlipidemic T2D	Turmeric (n = 11) Placebo (n = 10)	Three 700 mg of turmeric powder or placebo for eight weeks.	44.33 ± 1.23 44.22 ± 3.07	NR	0 (0) 0 (0)	Curcumin significantly decreased FBG and CRP.
Dastani et al., 2022 [51]	Randomized, double-blinded, placebo-controlled clinical trial	Iran	T2D	Nano-curcumin (n = 32) Placebo (n = 32)	Nano micelles containing curcumin ( 80 mg once daily) or placebo for three months.	60.00 ± 7.22 60.53 ± 10.55	26.15 ± 3.88 26.72 ± 3.9	11 (34.4) 14 (43.8)	Curcumin significantly decreased CRP.
de Sousa et al., 2021 [54]	Randomized, placebo-controlled, clinical trial	Brazil	T2D	Curcumin (n = 33) Placebo (n = 28)	Turmeric (500 mg), piperine (5 mg), or placebo once daily for four months.	63.2 ± 11.1 61.9 ± 11.0	29.66 ± 4.80 28.58 ± 5.00	10 (30.3) 4 (14.3)	Curcumin significantly reduced FBG and HbA1C.
Hosseini et al., 2024 [31]	Randomized, double-blind clinical controlled trial	Iran	T2DM and hypertriglyceridemia	Curcumin (n = 33) Placebo (n = 32)	One tablet of 505 mg curcumin-piperine daily or placebo for 12 weeks.	55.25 ± 7.46 56.17 ± 6.17	31.55 ± 6.42 30.55 ± 6.02	9 (25) 13 (36.1)	Curcumin significantly decreased FBG without effect on CRP.
Yaikwawong et al., 2024 [48]	Randomized, double-blinded, placebo-controlled clinical trial	Thailand	T2D	Curcumin (n = 113) Placebo (n = 114)	Three capsules of either 250 mg curcumin or a placebo twice daily for 12 months.	60.27 ± 8.82 62.26 ± 8.65	27.21 ± 3.93 26.76 ±4.06	62 (54.87) 54 (47.37)	Curcumin significantly decreased FBG, HbA1C, and CRP.

T2D: Type 2 diabetes, FBG: fasting blood glucose, HbA1c: glycated hemoglobin, CRP: C-reactive protein.

**Table 2:** Meta-regression analysis based on different moderators on FBG, HbA1C, and CRP in T2D on curcumin.

Outcomes	Moderators	Coefficient	95%CI	*p*-Value	I^2^ (%)
FBG [28,29,30,31,43,45,46,47,48,49,50,52,53,54,55,56]	Age	1.09	−5.14, 7.32	0.739	44.89
	Gender	−1.55	−8.88, 5.79	0.679	44.06
	Patient condition	−3.86	−10.22, 2.55	0.238	40.68
	Dose	−1.03	−4.72, 2.66	0.584	86.85
	Period of intervention	−0.93	−4.66, 6.52	0.745	87.16
	Risk of bias/quality	2.56	−3.22, 8.34	0.385	46.02
HbA1C [28,29,30,31,43,45,46,47,48,49,50,52,54,55,56]	Age	−0.29	−0.80, 0.23	0.251	39.7
	Gender	N/A	N/A	N/A	N/A
	Patient condition	−0.40	−1.21, 0.40	0.299	7.9
	Dose	0.05	−0.37, 0.47	0.788	15.3
	Period of intervention	0.22	−1.80, 0.61	0.251	6.1
	Risk of bias/quality	0.10	−0.36, 0.56	0.640	14.9
CRP [29,44,45,47,48,49,51,53,55]	Age	−0.53	−1.86, 0.79	0.381	44.5
	Gender	−2.74	−4.25, −1.24	0.003 **	0
	Patient condition	−0.59	−1.91, −0.71	0.325	42.8
	Dose	−0.48	−1.70, 0.75	0.395	44.1
	Duration of intervention	−0.51	−1.84, 0.81	0.395	44.8
	Risk of bias/quality	−0.53	−1.86, 0.79	0.381	44.5

T2D: Type 2 diabetes, FBG: fasting blood glucose, CRP: C-reactive protein., **, *p* < 0.01.

**Table 3:** Certainty of evidence profile according to recommendation, assessment, development, and evaluation (GRADE).

Certainty Assessment								No of Patients		Effect	
Outcome	No of Studies	Study Design	Risk of Bias	Inconsistency	Indirectness	Imprecision	Other Considerations	Curcumin	Placebo	Absolute, 95%CI	Certainty
FBG	16	Randomized trials	serious ^a^	serious ^b^	not serious	serious ^c^	strong association ^d,e,f^	637	629	MD 11.48 mg/dL lower (14.26 lower to 8.7 lower)	⨁⨁◯◯ Low ^a,b,c,d,e,f^
HbA1C	14	Randomized trials	serious ^a^	not serious ^g^	not serious	not serious ^h^	none ^d,f,i^	595	590	MD 0.54% lower (0.73 lower to 0.35 lower)	⨁⨁⨁◯ Moderate ^a,d,f,g,h,i^
CRP	10	Randomized trials	serious ^a^	serious ^b^	not serious	not serious ^h^	publication bias is strongly suspected all plausible residual confounding would reduce the demonstrated effect ^j,k,l^	352	350	SMD 0.59 SD lower (1.11 lower to 0.07 lower)	⨁⨁◯◯ Low ^a,b,h,j,k,l^

^a^. Although some trials had low risk in some of the domains, others had a high risk of bias in other domains, like randomization, allocation concealment, and blinding of participants and personnel. ^b^. An I-squared statistical value was greater than 50%, suggesting the variability in the trials, ^c^. The confidence interval (CI) of FBG was wider and thus less precision, ^d^. The funnel plot revealed no publication bias, further supported by Egger’s test (*p* > 0.05), ^e^. The effect size obtained is classified as moderate to large, according to Cohen’s d estimation, ^f^. No moderators were associated with the effect on the outcome, ^g^. An I-squared statistic value was 48%, deemed minimal heterogeneity, ^h^. The confidence interval was relatively narrow, indicating a fair precision, ^i^. The effect size was classified as moderate, based on Cohen’s d estimation, ^j^. The funnel plot showed evidence of publication bias, which was confirmed by Egger’s test (*p* < 0.05), ^k^. An effect estimate was deemed a moderate to large effect size according to Cohens d evaluation, ^l^. Through meta-regression, gender was significantly associated with reduced CRP.

## Data Availability

All data supporting this manuscript are provided in the Supplementary File.

## Supplementary Materials

The following supporting information can be downloaded at: https://www.mdpi.com/article/10.3390/nu16234177/s1, Supplementary File S1: PRISMA Checklist. Supplementary File S2: Screening procedure of studies. Supplementary File S3: Table S1: Search strategy adapted on databases; Table S2: Subgroup analyses evaluating curcumin on hyperglycemia and inflammation.
