# Supplementary material for: Curcumin Attenuates Hyperglycemia and Inflammation in Type 2 Diabetes Mellitus: Quantitative Analysis of Randomized Controlled Trial
Source: Nutrients. 2024 Nov 30;16(23):4177. doi: 10.3390/nu16234177 (PMC11644433; doi:10.3390/nu16234177)
Supplement: Supplementary file 1 [file nutrients-16-04177-s001.zip › Supplementary File S3_ Supplementay Tables.pdf]

Table S1: Search strategy adapted on databases

Database	Search	Number of records
PubMed	(((((Curcumin longa[MeSH Terms]) OR (curcumin[MeSH Terms])) OR (curcuma xanthorrhiza[MeSH Terms])) OR (turmeric[MeSH Terms])) OR (diferuloylmethane[MeSH Terms])) AND (type 2 diabetes[MeSH Terms]) Filters: Randomized Controlled Trial	33
Scopus	(TITLE-ABS-KEY (curcuma AND longa) OR TITLE-ABS-KEY (Curcumin) OR TITLE-ABS-KEY (curcuma AND xanthorrhiza) OR TITLE-ABS-KEY (diferuloylmethane) OR TITLE-ABS-KEY (turmeric) AND TITLE-ABS-KEY (type 2 diabetes AND mellitus) OR TITLE-ABS-KEY (type 2 diabetes))-	392
EBSCOhost	*Curcuma longa* OR curcumin OR turmeric AND type 2 diabetes mellitus AND randomized controlled trial	14
Web of Science	((((((TS=(curcuma longa)) OR TS=(curcuma xanthorrhiza)) OR TS=(curcumin)) OR TS=(turmeric)) OR TS=(diferuloylmethane)) AND TS=(type 2 diabetes mellitus)) AND TS=(randomized controlled trial)	114

Table S2: Subgroup analyses evaluating curcumin on hyperglycemia and inflammation

Outcome	Moderators	No of trials	Effect size	95%CI	*P*	I^2 of subgroup difference
FBG	Older than 50 years	13	–11.89[b]	–7.90, –20.02	<0.001	38.3
	Younger than 50 years	3	–10.87[b]	–29.6, 7.86	0.130	75.2
	Both	15	–11.26[b]	–15.05, –7.46	<0.001	41.8
	Females	1	–12.83[b]	N/A	N/A	N/A

	T2D	13	–10.62[b]	–14.30,–6.94	<0.001	38.5
	T2D + hyperlipidemia	3	–15.54[b]	–32.63, 1.56	0.060	34.6
	Less than 1000 mg	10	–10.28[b]	–15.52, –5.056	<0.001	40.5
	More than 1000 mg	6	–12.21[b]	–16.75, –7.67	0.002	89.7
	Less or ten weeks	5	–12.13[b]	–17.98,–6.28	<0.001	46.9
	Longer than ten weeks	11	–11.26[b]	–15.40, –7.12	0.002	89.7
HbA1C	Older than 50 years	12	–0.49[b]	–0.71, –0.28	<.001	45.1
	Younger than 50 years	2	–0.76[b]	–1.99, 0.47	0.08	0
	Both	14	-0.54[b]	–0.73, –0.35	<0.001	45.7
	Females	0	N/A	N/A	N/A	N/A
	T2D	13	–0.52[b]	–0.71, –0.32	<0.001	44.8
	T2D + hyperlipidemia	1	–0.92[b]	N/A	N/A	N/A
	Less than 1000 mg	10	–0.53[b]	–0.84, –0.22	0.004	55
	More than 1000 mg	4	–0.54[b]	–0.86, –0.23	0.012	23.3
	Less or ten weeks	4	–0.42[b]	–0.89, 0.06	0.70	60.7
	Longer than ten weeks	10	–0.63[b]	–0.88, –0.38	<0.001	38.9
CRP	Older than 50 years	11	–0.48[a]	–0.89, –0.07	0.029	67.8
	Younger than 50 years	3	–1.20[a]	–5.11, 2.72	0.319	90.1
	Both	13	–0.44[a]	–0.76, –0.12	0.13	62.4
	Females	1	–3.21[a]	N/A	N/A	N/A
	T2D	12	–0.46[a]	–0.88, –0.04	0.036	69.5

	T2D + hyperlipidemia	2	–1.23[a]	–5.06, 2.60	0.301	88.7
	Less than 1000 mg	10	–0.47[a]	–0.87, –0.01	–0.44	64.5
	More than 1000 mg	4	–1.03[a]	–3.11, 1.05	0.214	87.4
	Less or 10 weeks	3	–0.47[a]	–0.83, –0.10	–0.831	60.9
	Longer than 10 weeks	11	–1.19[a]	–5.23, 2.85	0.333	76.9

a: data reported as mean difference, b: data reported as standardized mean difference
